# *QuickStats:* Rate* of Emergency Department Visits,^†^ by Homeless Status^§^ — National Hospital Ambulatory Medical Care Survey, United States, 2010–2021

**DOI:** 10.15585/mmwr.mm7242a6

**Published:** 2023-10-20

**Figure:**
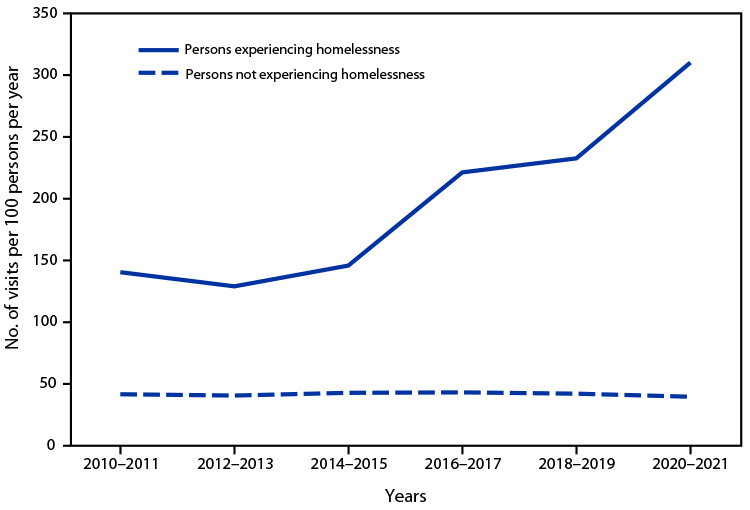
The rate of visits to hospital emergency departments by persons experiencing homelessness increased from an estimated 141 visits per 100 persons per year during 2010–2011 to 310 during 2020–2021. Rates increased during 2016–2017 compared with 2014–2015, and again during 2020–2021 compared with 2018–2019. Visit rates for persons not experiencing homelessness did not vary significantly across years, ranging from 42 visits per 100 persons per year during 2010–2011 to 40 during 2020–2021. Visit rates for persons experiencing homelessness were higher than rates for persons not experiencing homelessness in all years.

**Source:** National Center for Health Statistics, National Hospital Ambulatory Medical Care Survey, 2010–2021. https://www.cdc.gov/nchs/ahcd/ ahcd_questionnaires.htm

